# Digital–environmental habitus of families in England in times of pandemic

**DOI:** 10.1177/14614448221146716

**Published:** 2023-01-12

**Authors:** Maria Laura Ruiu, Gabriele Ruiu, Massimo Ragnedda

**Affiliations:** Northumbria University, UK; University of Sassari, Italy; Northumbria University, UK

**Keywords:** Climate awareness, climate change, digital technology, habitus

## Abstract

This article uses adopts a revised version of the concept of techno-environmental habitus to investigate and make sense of the differentiation among digital technology users’ attitudes towards the environment in England. Digital–environmental habitus refers to the combination of structural determinants (existing background) and the metabolised increased use of digital technologies in people’s everyday life that also interacts with individual environmental attitudes. The results of a national survey among English parents between 20 and 55 years suggest that parents’ education levels, gender, age and income play a role in increasing their awareness about the environmental-friendly use of digital technologies. This study shows that the digital–environmental habitus of parents in England is layered according to the combination of existing socioeconomic traits and individual capacity and willingness to adapt to a drastic increase in both the use of digital technologies (due to the social distancing imposed by the pandemic) and environmental degradation.

## Introduction

This article draws upon and revises the concept of techno-environmental habitus (Ruiu et al., 2021) to interpret the differentiation among digital technology users’ attitudes towards the environment to lower their carbon footprint. Habitus might be understood as the lens through which individuals interpret social reality (and act accordingly) thanks to ‘a set of perceptive patterns and expectations’ (Piroddi, 2021) and it is layered through the unconscious acquisition of cultural, economic and social backgrounds (structural determinants) from the early stages of life (Bourdieu, 1977), and the continuous transformation/adaptation to new contexts (Abrahams and Ingram, 2013; Bourdieu, 2002; Di Maggio, 1979; Reay, 2004). Drawing on this definition, digital–environmental habitus refers to the combination of structural determinants (existing background) and the metabolised increased use of digital technologies in people’s everyday life that also reflects individual environmental attitudes. In this context, ‘environment’ refers to all the natural components (living and non-living elements) and their interaction with human life, whereas digital technology specifically refers to Information Communications Technologies (ICTs). A previous study conducted by Ruiu et al. (2021) in the United Kingdom suggested that the connection between sociodemographic traits and environmental attitudes should also take into account the use of technologies as a choice to reduce the individual impact on the environment. This is because the passage to a pervasive technological use might trigger an alteration of what Bourdieu defines as habitus (Bourdieu, 1977, 1990; Bourdieu and Wacquant, 1992).

Previous works on techno-environmental habitus have mainly focused on the relationship between media users and predispositions to climate change (CC). Several studies have focused on the use of the media and specific aspects of environmental changes, such as CC understanding, for example, focusing on social media discussions (see, for example, Connor et al., 2016; Diehl et al., 2021; Gladston and Wing, 2019; Mavrodieva et al., 2019; Pearce et al., 2019; Shah et al., 2021), for instance, underlined how users’ pro-environmental behaviours are positively influenced by exposure to information about CC on social media. However, there is still a need to investigate how the use of digital technologies reflects people’s overall environmental dispositions.

The originality of this work lies in exploring the interaction between digital techno-use and environmental dispositions, revealing different digital–environmental habitus, which may either facilitate or hinder both individual and collective sustainable actions to protect the environment. Moreover, the recovery from repeated lockdowns owing to the COVID-19 pandemic represents the ideal moment to study the effects of technological acceleration and people’s use of ICTs in line with their environmental predispositions. The ecological and social crisis on one hand, and digitalization on the other, enables the convergence of global and common goals and priorities for a sustainable future. This is also an ideal moment to intervene to educate users on the sustainable uses of digital technologies to limit potential negative impacts on the environment. Since the literature optimistically interprets the socioecological context of the pandemic as an opportunity to develop a new sustainable global culture (Galvani et al., 2020), the passage from the COVID-19 crisis to the post-pandemic represents an unprecedented opportunity to study the digital–environmental habitus. In this way, the pandemic issue may be viewed as a ‘dialectical conflict’ between people’s ‘old’ and ‘new’ lifestyles (Bourdieu, 1977, 1990; Bourdieu and Wacquant, 1992), with the potential to alter the eco-habitus integrating digital behaviours into daily life.

It also coincides with increasing media attention given to the CC threat in the United Kingdom, thanks to the UK Government’s commitment to net-zero carbon by 2050, the Greening Government Commitments 2020–2025, the UN Sustainable Development Goals (SDGs) and the 25-Year Environment Plan (Department for Environment Food & Rural Affairs, 2020) and the United Nations CC Conference in Glasgow (November 2021). Therefore, the study of the combination of environmental and techno-digital attitudes might create favourable conditions for the institutional frame to redirect the techno-digital orientation of society towards pro-environmental practice even after the COVID-19 crisis is over.

This article is based on an online survey with around 2000 parents of children aged at least 5 years old who are attending school. A total of 1984 valid responses were included in the present study. The survey focuses on families with children given the documented orientation of this group to pay more attention to environmental issues. For example, a study conducted in Innsbruck (Fornwagner and Hauser, 2021) showed that parents are more likely to engage in voluntary climate action. Studies on ecological awareness have revealed that children of all ages have an impact on their parent’s behaviour and attitudes (Carrete et al., 2012; Watne et al., 2011). Also, media narratives were often found to represent parents as eco-anxious through parenthood (Benoit et al., 2022). Moreover, in 2021, the Office for National Statistics found that adults in Great Britain were mostly worried about the future of the environment in relation to their families and future generations. At the same time, Milfont et al. (2020) found that becoming a parent might positively influence beliefs in the reality of CC but does not affect environmental attitudes. Additional studies found no associations between being parents and both CC concerns (McCright, 2010; Sundblad et al., 2007) or environmental attitudes (Torgler et al., 2008). Thomas et al. (2018) also found that becoming a parent for the first time does not significantly change parents’ attitudes towards the environment. Given this variegated picture, one aspect that still needs to be searched relates to the age of children. Since the present study is interested in identifying some traits that might characterise an environmentally oriented use of digital technologies, the sample consists of parents who are also digital users.

Therefore, the relationship between parenting and both environmental awareness and behaviour is still an emergent field of study that has predominantly focused on the relationship between being a parent and CC. This relationship has been explored by looking at the urgency of action (Cripps, 2017), parents’ anxiety about CC (Ekholm and Olofsson, 2017; Gaziulusoy, 2020) and how parenting interacts with the wider sociocultural context and impacts specific environmental issues (Burton and Farstad, 2020).

Directly connected to the conflictual results that emerged in the literature, the first hypothesis explores if the age of the children might contribute to explaining parents’ digital–environmental habitus:

*H1.* The age of children impacts the digital–environmental habitus of families.

The literature shows contrasting results concerning the sociodemographic effects on environmentally oriented behaviours by suggesting that they cannot be the unique factors that segment individual environmental behaviours and attitudes towards the environment (Sargisson et al., 2020). However, several studies (Boeve-de Pauw and Van Petegem, 2010; Casaló and Escario, 2018; Franzen and Vogl, 2013; Lee et al., 2015) identified an influence of sociodemographic traits on individuals’ environmental attitudes, and some sociodemographic variables such as age, income and education have been found to influence digital experience by impacting both access and competencies of users (Calderon et al., 2022; Ragnedda et al., 2020). Moreover, Ruiu et al. (2021) have shown a relationship between cultural capital and techno-environmental habitus. Therefore, the second hypothesis investigates the relationship between some sociodemographic traits and digital–environmental habitus:

*H2.* Sociodemographic characteristics (such as education, age, gender, parents’ status and income) impact the digital–environmental habitus.

Finally, a previous study conducted by Ruiu et al (2021) investigated the stratification of the techno-environmental habitus according to media use and CC attitudes in the United Kingdom through an online survey of a sample of the UK population (1013 respondents). They found that respondents were associated with ‘advocacy’ positions (around 44% of the variance), and characteristics that can be associated with CC ‘scepticism’ opinions (around 18% of the variance). However, individuals’ environmental predispositions might not be entirely explained by their perception of CC. The definition of climate ‘as the mean physical state of the climatic system, which is constituted by atmosphere, hydrosphere, cryosphere, lithosphere and biosphere, which are intimately interconnected’ (Lucarini, 2002: 413) emphasises that climate is a specific component of the natural environment. However, the perception of CC does not necessarily correspond to that of the environment. It is possible to be environmentally friendly (and, for example, believe in the importance of preserving and protecting the environment), but sceptical about some aspects of CC.

In fact, some studies found that CC sceptics might also hold pro-environmental views (Haltinner and Sarathchandra, 2020, 2022), suggesting that CC perception might not adequately capture individuals’ attitudes towards the environment. Therefore, a third hypothesis aims to investigate the relation between digital–environmental orientations and belief in CC:

*H3.* The digital–environmental habitus is not necessarily connected to specific beliefs in CC.

The following section reviews the literature on the concept of habitus and how it can be applied to the combination of digital–environmental attitudes. The third section outlines the strategy followed to collect and analyse the data, while the fourth section presents and discusses the results of the analysis. Finally, some discussions and conclusions will be drawn.

## Literature review

The concept of techno-environmental habitus is a valuable tool for studying the stratified nature of techno-users’ predispositions towards the environment (Ruiu et al., 2021) and in turn formulating suggestions for policies that simultaneously consider technology use and the environment. This study updates this concept to tailor it to the techno-digital acceleration due to the COVID-19 pandemic that forced services, resources and opportunities to migrate online, making the use of digital technologies an indispensable part of everyday life. The ability of digital technology to alter lives, economies, cultures and societies that characterises the ‘Information Age’ (Castells, 2010), became even more evident with the COVID-19 pandemic that has enhanced the ongoing digitalization of society, cementing digital as the new normal. The digital revolution is directly relevant to social behaviours and organisations focused on protecting the environment and lowering carbon emissions. Digital technology has a growing impact on how people see, contemplate and interact with nature (Kahn, 2011), and it may aid in lessening the anthropogenic factors contributing to CC. Therefore, this article refers to the digital–environmental habitus as the combination of structural determinants (existing background) and the metabolised increased use of digital technologies in people’s everyday life that also interacts with individual environmental attitudes.

Habitus is layered through the unconscious acquisition of cultural, economic and social backgrounds (structural determinants) from the early stages of life (Bourdieu, 1977), and the continuous transformation/adaptation to new contexts (Abrahams and Ingram, 2013; Bourdieu, 2002; Di Maggio, 1979; Reay, 2004). Habitus is layered across society (Sterne, 2003) because it results from the internalised capitals, which are acquired starting from the early stages of life, and the contact with external fields (Bourdieu, 2002; Di Maggio, 1979; Reay, 2004). The field can be interpreted as the operational space of social actors, a ‘space of social forces and struggles’ (Bourdieu and Wacquant, 1992) between different social actors’ interests. The value of habitus can be associated with the individual unconscious capacity (Bourdieu, 1990, 1977) of people to interpret social reality (Bourdieu and Chartier, 2015; Elam, 2008; Hughes and Paterson, 2017). Therefore, habitus is neither entirely defined by external determinants nor by the individual agency (Bourdieu, 1990; Crossley, 2002). It results from subjective (personal experience of a diverse combination of structural constraints and context of action) and collective dimensions (dispositions and attitudes acquired in social groups) (Bourdieu and Wacquant, 1992; Ingram, 2011; Schmidt, 1997). Habitus has a bridge function between individual actions and the context for these actions (field) (Brulle and Norgaard, 2019; Ruiu et al., 2021). The ‘dialectical confrontation’ (Ruiu et al., 2021) between existing habitus and its capacity to adapt to social transformation (e.g. increased use of digital technologies due to the pandemic) might be progressively solved thanks to the capacity of habitus to unconsciously metabolise new stimuli.

Some studies focused on the adaptive capacity of habitus in relation to both technology use and environmental dispositions. Further evolutions of this concept and its application have developed two classifications of habitus in terms of ‘ecological habitus’ (or ‘eco-habitus’) (Adams, 2012; Eriksen, 2013; Haluza-DeLay, 2008; Kasper, 2009; Kirby, 2017) and ‘digital habitus’. Eco-habitus has been defined as the set of social and ecological practices developed according to the context of action (Haluza-DeLay, 2008; Smith, 2001), which influence the tastes, practices and dispositions of consumers (Carfagna et al., 2014) to protect the environment (Maguire, 2016). A study in the Czech Republic (Pelikán et al., 2017) found that the ecological habitus is reproduced intergenerationally from parents to children. Similar results were also found in Finland (Leppänen et al., 2012), where children reproduce their parents’ attitudes towards the environment. These studies are particularly useful for the foundation of the present article, which aims to investigate the digital–environmental habitus of parents.

Costa et al. (2019) also identify some key institutions that contribute to increasing the rapid transformation of society, one of which is represented by the Internet. The authors refer to a continuous redefinition of an individual’s habitus according to the field’s evolution, which, in turn, can lead to a shift in attitude and practice. Some scholars refer to technological habitus as the interaction between collective practice absorbed by the habitus and individual action (Costa, 2014; Kvasny, 2005). More specifically, digital habitus might be interpreted as a continuous engagement with digital technologies (Richardson, 2015) that differentiates users from previous generations (Zevenbergen, 2007). This concept has been especially used in the context of higher education to explain the individual capacity to ‘evolve’, thanks to the use of diverse types of technology and the individual pre-existing ability to benefit from their use (Czerniewicz and Brown, 2013).

Finally, Ruiu et al. (2021) introduced the concept of ‘Techno-environmental habitus,’ which combines existing background and individual capacity to adapt to both a drastic increase in the use of technologies (due to the social distancing imposed by the pandemic) and the increasing perception of CC as a threat in the United Kingdom (Department for Business, Energy & Industrial Strategy, 2021). This study referred to the interconnection between CC and technology use, because of the frequent requests by the public regarding potential interconnections between CC and COVID-19 to scientists and policymakers during the pandemic (Bernstein, 2020; WHO, 2020). Slightly different from Ruiu et al.’s definition, which exclusively connects the use of technologies to CC awareness, the present article refers to digital–environmental habitus as the use of digital technology—specifically ICTs—in a broader environmental-oriented way, not only connected to CC. The present study reboots Bourdieu’s relational notions of habitus, digital technologies and environment by revealing how these notions have evolved to fit with the new digital society and contemporary techno-digital culture. Therefore, digital–environmental habitus includes both individual perceptions of the impacts of using digital technologies on the environment (digital carbon footprint) and behavioural responses. Nevertheless, the restrictions imposed by the pandemic created the conditions for digital technologies to proliferate in multiple sectors and contribute to generating impacts on the environment (Elavarasan and Pugazhendhi, 2020), therefore, giving individuals the chance to reevaluate societal behaviours more sustainably. The increasing media coverage of environmental-related issues coupled with the COVID-19 crisis might represent the appropriate moment to activate processes of context-dependent reflexivity (Pedersen, 2000; Shove, 2004). However, the relationship between the possession of digital tools and digital competencies and the promotion of environmental engagement has been scarcely considered by the literature. Extensive attention has been devoted to studying media products (mainly news media) as a predictor of environmental behaviour (see, for example, Östman, 2014) and awareness (Arlt et al., 2011; Shah et al., 2021; Zhang and Skoric, 2018). On one hand, research into the digital economy has shown that digital skills empower consumers to make green choices (Gazzola et al., 2017); on the other hand, it does not consider the preference for using digital technologies (instead of, for example, materially accessing a service) as a reflexive choice to limit the physical impact on the environment with regard to certain activities.

Moreover, the COVID-19 crisis might have created ‘opportunities’ resulting from the combination of structural/contextual (such as opportunities to work from home, restrictive rules and existing individuals’ socio, cultural and economic backgrounds) and individual traits (predisposition towards the environment and the adoption of digital technologies) that might have made individuals realise that maintaining some digital components and behaviours in their everyday life might not affect their comfort while benefitting the environment. The fragmentation of experiences might, indeed, cause either a rejection of the original habitus (Wentworth and Peterson, 2001) or a ‘chameleon’ transformative capacity (Abrahams and Ingram, 2013). In this evolving context, following Piroddi (2021), habitus can be used as an analytical tool for identifying the social factors that shape interrelationships and reciprocal recognition and contribute towards their sedimentation. Therefore, the recovery from the COVID-19 pandemic might represent either a catalyst for sensitising users to benefit from digital technologies in respect of the environment or can provide opportunities to develop multiple different behavioural patterns. In this sense, when opposing forces are at play (between past and new fields) a constellation of new habitus configurations might be generated, which might be difficult to reorient towards an environmentally friendly use of digital technologies once they are consolidated. The COVID-19 recovery, therefore, might be a chance to help avoid metabolising the use of digital technology in everyday life without considering the environmental consequences of the digital growth of our societies.

## Methods

### Design

Given the nature of the digital–environmental habitus, which following the literature review can be interpreted as the result of ‘opposing’ forces represented by what users know/believe and how they understand and act in a new field, the present work refers to the concept as a combination of existing techno-digital dispositions and environmental dispositions and how they combine in a field in which both digital technology uses and environmental degradation have been rapidly accelerating. It is generally recognised that sensitisation campaigns that aim to impact individual behaviour tend to be more effective if they are tailored to specific groups (Steg, 2008). This is the reason why this article focuses on a specific segment of the English population to explore some traits that might be helpful to predict digital–environmental attitudes in a moment of drastic techno-digital acceleration such as that caused by the COVID-19 pandemic.

### Sample

The present survey focused on families with children given that some studies point towards the orientation of this group to environmental issues (Fornwagner and Hauser, 2021). A randomised stratified sample of English online users between 20 and 55 years old with children at school was used (N = 1984) and was collected in March 2022 by *Lucid* an online research market company. The sample was stratified on age (1%, 20–24 years old; 32% between 25 and 34; 40% between 35 and 44; and 27% between 45 and 55), education (4% with some high school; 21% with a high school diploma; 25% with some credit college; 5% with no degree; 33% with a degree; 10% with a master’s degree; and 2% with a PhD), gender (51% female and 49% male respondents), family income (6% of families under £10k; 25% between £11 and £25k; 42% between £26 and £50k; 23% between £51 and £100k; and 4% over £100k) and parents’ status (74% of parents living together and 26% between single parents, widowed, divorced and separated).

The survey is based on those who use digital technologies, and specifically ICTs, to explore how the combination of the use of digital technologies in everyday life and environmental predispositions interact with existing backgrounds and CC perception. The online survey used software that checked for missing responses and then prompted users to respond. The survey was pilot tested with 20 Internet users over two rounds. Amendments were made based on the feedback provided. The average time required to complete the survey was 25 minutes.

### Measures and analysis

Following the definition of digital–environmental habitus, the first step consisted of creating a digital–environmental habitus Index that consists of both awareness and behaviours towards the use of digital technologies to minimise the individual impact on the environment. A two-step factorial analysis (FA) was performed. The first dimension related to behaviours was investigated through an *ad hoc* set of items reported in Table 1 by asking respondents to express their agreement on a scale from 0 (totally disagree) to 10 (totally agree) about the relationship between their everyday use of digital technologies and their impact on the environment. The FA shows the emergence of two components, which were extracted based on an eigenvalue higher than 1. The first component is purely related to a vocation to use digital technologies in a way that also ‘protects’ the environment (and explains 39% of the variance), and the second component shows environmentally friendly uses of digital technologies when other practical benefits are present (and explains 16% of the variance). Items related to unsubscribing from automatically generated newsletters and condensation of messages in emails have a positive association with this component.

**Table 1.** Behaviour dimensions of respondents’ digital–environmental habitus.

	Environment-oriented	Benefit-oriented
I prefer to meet people online rather than face to face (e.g. to limit my physical movements and reduce my impact on the environment)	.623	.035
I try to condensate as much information as I can in one email/message	.168	.719
I limit my online activities (e.g. searches, watching YouTube videos, posting on social media) because they hurt the environment	.836	.024
I unsubscribe from automatically generated newsletters	.028	.811
I order online only if I need multiple items	.625	.268
I avoid express delivery (1 day delivery)	.559	.183
I check if the businesses are respectful of the environment before ordering online	.847	−.015
If I use on-demand video services or other streaming services, I make sure that videos are in low resolution	.847	−.015

Extraction method: principal component analysis; Rotation method: varimax with Kaiser normalisation.

Following the same procedure, an FA was performed to explore a set of items asking respondents to reflect on their awareness of the impact of digital technologies on the environment. In this case, responses converged in a unique component (which explains 49% of the variance) that emphasises a tendency to reflect on the impact of technologies on the environment in terms of both benefits and drawbacks (Table 2).

**Table 2.** Awareness dimension of the digital–environmental habitus.

	Digital–environmental awareness
I get most of my knowledge on climate change on the Internet	.611
I find myself reflecting on how my technological behaviour may impact the environment	.808
I know that technologies are harmful to the environment	.746
Online shopping is more eco-friendly than in-store shopping	.601

Extraction method: principal component analysis.

Finally, to develop an Index of digital–environmental habitus, both dimensions (Behavioural and Awareness) were summarised in a new variable (digital–environmental habitus) through an FA (Table 3), which explains 52% of the variance of the environmental orientation of respondents. This method was validated by considering an alternative procedure consisting of summing up the new emerging factors (related to both awareness and behaviour). However, the results were similar when the new variable was included in further analyses, and the FA method was considered appropriate.

**Table 3.** Digital–environmental habitus.

	Digital–environmental habitus
Environment-oriented digital–environmental habitus	.807
Benefit-oriented digital–environmental habitus	.360
Environmental awareness digital–environmental habitus	.887

Extraction method: principal component analysis.

The new continuous variable generated from the FA was used to investigate the hypotheses proposed in the introduction of this article through a multiple linear regression that tested three different models. The age group of children, parents’ status, age of parents, gender of respondents, family income and education were used as predictors of digital–environmental habitus to test the first two hypotheses.

H3 was investigated by adding to the model two variables related to scepticism and realism around CC. The latter two variables were generated by asking respondents to provide their level of agreement on a scale from 0 (totally disagree) to 10 (totally agree) about their knowledge and beliefs of CC. The items included in this set aimed to investigate respondents’ awareness of the causes and consequences of CC, as well as behaviours and actions needed to limit the problem (Table 4). An FA was performed, and it generated two components, which are related to ‘scepticism’ and ‘realism’ around CC (Table 4). The first component includes items that emphasise scepticism around several aspects of CC such as uncertainty about the reality of the phenomenon, lack of scientific evidence and agreement among scientists, exaggeration of the consequences and no need to act to contain the problem. The second component shows a convincement about scientific agreement around the reality of the phenomenon, which is frightening, especially because of its ‘catastrophic’ consequences, and the need for individual actions as well as regulation of society’s behaviours.

**Table 4.** Perception of climate change among respondents.

	Scepticism	Realism
We can all do our bit to reduce the effects of climate change	−.304	.628
People should be made to reduce their energy consumption if it reduces climate change	−.073	.740
Climate change will improve our weather	.523	.222
Climate change is just a natural fluctuation in the earth’s temperatures	.777	−.179
It is already too late to do anything about climate change	.695	–.024
Climate change is something that frightens me	−.011	.771
I am uncertain about whether climate change is really happening	.821	–.202
Radical changes to society are needed to tackle climate change	−.167	.793
The evidence for climate change is unreliable	.820	−.240
Claims that human activities are changing the climate are exaggerated	.826	−.264
If I come across information about climate change I will tend to look at it	.015	.743
The effects of climate change are likely to be catastrophic	−.135	.802
Nothing I do makes any difference to climate change one way or another	.768	−.234
Experts are agreed that climate change is a real problem	−.260	.706

Extraction method: principal component analysis; Rotation method: varimax with Kaiser normalisation.

## Results and discussion

The three hypotheses were investigated through multiple linear regressions based on three models (see Table 5). The number of children was excluded from the model because it did not have any significant contribution to explaining the variance of the digital environmental habitus. The first model considers children’s age group (H1) and parent status (either living or not living in the same household) as predictors of digital–environmental habitus. This model suggests that if the parents live together their digital–environmental habitus increases. A reasonable explanation for this might be that single parents may have different concerns to worry about than the eco-friendly use of digital technologies. Downey and colleagues (Downey, 2005; Downey and Hawkins, 2008; Downey et al., 2017) have repeatedly shown that single parents are more likely to live in neighbourhoods with a higher toxic concentration of pollutants than other types of families. While this might be associated with a tendency for single parents to have lower incomes, it also suggests that other factors represent priorities for this group.

**Table 5.** Multiple linear regressions between environment-oriented uses of technologies and existing traits.

Model		Unstandardised coefficients		Sig.	Collinearity statistics	
		*B*	SE		Tolerance	VIF
1	Constant	−.095	.110	.388		
	Children age group 1–5	−.070	.039	.072	.862	1.161
	Children age group 6–11	−.093	.039	.016	.796	1.257
	Children age group 12–17	−.066	.038	.079	.771	1.297
	Children age group 18–25	−.159	.057	.006	.872	1.147
	Children age group > 25	.060	.116	.601	.925	1.081
	Parent status (both parents living together)	.152	.057	.008	.965	1.036
2	Constant	.703	.209	.001		
	Children age group 1–5	−.094	.041	.021	.745	1.343
	Children age group 6–11	−.110	.038	.004	.774	1.292
	Children age group 12–17	−.021	.038	.577	.739	1.352
	Children age group 18–25	−.071	.057	.215	.832	1.202
	Children age group > 25	.161	.114	.158	.905	1.105
	Parent status (both parents living together)	.048	.060	.428	.818	1.222
	Mean age of parents	−.015	.004	.000	.674	1.483
	Family income	−.037	.037	.312	.728	1.374
	Gender (female)	−.255	.049	.000	.926	1.080
	Family education	.163	.027	.000	.773	1.294
3	Constant	.411	.116	.000		
	Children age group 1–5	−.041	.022	.065	.742	1.349
	Children age group 6–11	−.029	.021	.168	.771	1.297
	Children age group 12–17	−.005	.021	.819	.737	1.357
	Children age group 18–25	.002	.031	.953	.829	1.207
	Children age group > 25	.077	.063	.220	.903	1.107
	Parent status (both parents living together)	−.035	.033	.292	.817	1.225
	Mean age of parents	−.004	.002	.056	.656	1.525
	Family income	−.053	.020	.009	.724	1.381
	Gender	−.184	.027	.000	.913	1.096
	Family education	.090	.015	.000	.765	1.306
	Scepticism	.529	.013	.000	.955	1.047
	Realism	.631	.013	.000	.967	1.035

VIF: Variance Inflation Factor.

Model 1: *R*^2^ .008, Sig. .004; Model 2: *R*^2^ .055, Sig. < .001; Model 3: *R*^2^ .717, Sig. .000.

Two children’s age groups play a significant role in predicting the increase of digital–environmental habitus. The presence of children in the age groups 6–11 and 18–25 predicts a decrease in digital–environmental habitus. Even if other age groups are not significant predictors of digital–environmental habitus, they are, however, negatively associated with it. This might be interpreted in light of the literature that highlights how parents’ behaviours (including consumption and purchase decisions) are likely to be shaped by prioritising children’s well-being instead of other factors (such as the environment) compared with their previous status (Thomas et al., 2018; Thompson et al., 2011). H1 related to the influence of children’s age is only partially confirmed. Model 2 shows that when taking into account other sociodemographic traits of the family, the younger the children are the least parents tend to pay attention to environmentally friendly uses of digital technologies. Moreover, in all three models having children show that there is always a negative link with the digital–environmental habitus. As suggested in the introduction of this article, Fornwagner and Hauser (2021) found that parents tend to engage more in climate voluntary actions; however, the authors specifically refer to CC and not environmental orientations in general. By contrast, considering the environment in general, parents of younger children might be more concerned about other imminent threats rather than environmental problems that might be caused by digital technologies.

Model 2 adds several sociodemographic variables (H2) as predictors of digital–environmental habitus. The variable family education was created by attributing an increasing score to the education levels of parents (from 1 = no diploma to 6 = PhD) and calculating the average value of the education qualifications of both parents. This is also to avoid homogamy which can lead to a serious problem in the standard errors due to the correlation between the qualification of the two members of the couple. The variable age is inserted as the mean of the parents’ age.

This model shows that those who have younger children (1–11 years old) tend to be less concerned with the eco-friendly use of digital technologies. In line with the literature, parents’ age plays a role (despite a negligible influence) in negatively influencing the eco-friendly use of digital technologies and higher family education also predicts higher digital–environmental habitus. This is a valuable result in policy terms, given it further reinforces previous findings that have highlighted how teenagers with well-educated parents tend to have an interest in the environment (Braun et al., 2018; Stevenson et al., 2017).

In this model, the parental status loses its explicative power. In contrast to the ‘Parenthood Status hypothesis’ (Blocker and Eckberg, 1997) based on the assumption that mothers become more concerned for the environment (due to an increasing concern for children’s health), whereas fathers are less concerned for the environment (due to a concern for material well-being), women with children are associated with lower values of digital–environmental habitus compared with men.

In terms of gender differences, this result should be taken cautiously. To identify potential explanations for this difference, we looked at the association between the gender of respondents and those parents who took care of children’s schooling during the pandemic and between gender and their employment in a technology-related job. Tables 6 and 7 show a higher number of women who took care of their children during lookdowns compared with men, as well as a lower number of women who work in the technological field. This might contribute towards lower attention to an eco-friendly use/awareness of digital technologies for women. Female parents might have been, indeed, less exposed to digital technology and therefore had fewer chances to reflect on their environmental impact (or use digital technologies in an eco-friendly way). The literature pays more attention to female parents who might be more concerned about the environment (Davidson and Freudenburg, 1996; Thomas et al., 2018) than male parents (Blocker and Eckberg, 1989). Therefore, this result is interesting in light of potential concerns among male parents about adopting eco-friendly digital behaviours.

**Table 6.** Crosstabulation between gender and parent taking care of children during pandemic homeschooling.

During the lockdowns who did take care of your children while they were homeschooling?						
	Myself	The other parent	Both parents	Someone else	My children attended school	Total
Male	247	200	477	11	43	978
Female	632	44	235	25	70	1006
Total	881	244	714	36	113	1984

Gender-dependent η: .423.

**Table 7.** Crosstabulation between gender and type of job.

	Telecomm Technology/ Media	Other	Total
Male	99	879	978
Female	20	986	1006
Total	119	1865	1984

Gender dependent η: .174.

Finally, family income does not play a significant role in predicting the dependent variable.

H2 related to the influence of sociodemographic traits is partially confirmed by showing that parents’ age, gender and education are predictors of digital–environmental awareness. However, family incomes are not a significant predictor and even when they become significant in Model 3, they still have a limited negative effect (B = −.053).

The first two models only explain a small percentage of the variance of digital–environmental habitus. By contrast, adding sceptic and realist orientations to CC to the model (H3), this percentage drastically increases to 71%. This model shows that, when these two variables are considered, the effect of the age of the children no longer has a significant contribution to explaining the variance of their parents’ digital–environmental habitus. The male gender of the respondent still tends to predict greater values of digital–environmental habitus. Both orientations (sceptic and realist) have a positive significant contribution to predicting digital–environmental habitus, suggesting that environmental-friendly attitudes are positively connected to both realism and scepticism around CC. Therefore, those who believe that CC exists also tend to make use of digital technologies environmentally oriented to reduce their digital carbon footprint. However, also those who do not believe in CC might do the same. Therefore, H3 is confirmed. As Model 3 shows, both beliefs in CC (either scepticism or realism) positively predict digital–environmental habitus by suggesting that those who have higher degrees of digital–environmental habitus hold opinions on CC but are not necessarily in line with mainstream science. The correlation matrix generated with the multiple linear regression (not reported here) also showed that family education only has a significant influence on realism but does not impact scepticism. Therefore, even though higher qualifications are associated with both beliefs in climate science and higher degrees of digital–environmental habitus, those parents who tend to use digital technologies in line with environmental principles are not necessarily CC supporters. This is an interesting finding that suggests that concern for CC should not be used as a proxy for environmental concern, at least when investigating the digital habits of families.

## Conclusion

This study showed that the digital–environmental habitus of parents, which is intended as the combination of existing backgrounds and individual capacity to adapt to a drastic increase in the use of digital technologies (due to the social distancing imposed by the pandemic) and environmental degradation, is multifaced and dependent on several different factors.

Habitus is a complex construct that ensembles metabolised analytical schemes and behaviours (depending on pre-existing conditions), the rules of the field of social action, but also beliefs and expectations of the outcomes that are produced by specific actions in a field (Piroddi, 2021). Therefore, in the post-lockdown realm, while it is important to define the rules of the field (increased use of digital technologies in everyday life coupled with a dramatic increase in environmental degradation) to orient the use of digital technologies to minimise the individual impacts on the environment, it is also relevant to pay attention to what individuals expect to get from their actions (Bourdieu, 2000). Therefore, this article included both existing backgrounds and expected results from using digital technologies and their environmental impacts. The recovery phase from the pandemic might represent the first step to enhancing processes of collective transformation that involve both the individual and system levels. The digital acceleration due to the COVID-19 pandemic had a disruptive impact on how individuals live at the global and local levels (Salama, 2020), changing their habits and attitude towards the environment. Innovation and digitalization are potent tools for the achievement of a better society. However, to meet the SDGs by 2030, it is necessary to readdress the digital revolution in a more sustainable way (Sparviero and Ragnedda, 2021). The routinisation of digital technologies into everyday life along with an eco-friendly use of digital technologies is a way to support sustainable development, address the environmental problem, and advance a sustainable future.

This article identified some factors that can help understand digital technology practices in an eco-friendly light among English parents. The conceptual framework used in this article has shown potential in supporting the investigation of the intersection between individual choices and contextual opportunities. However, further research is needed to understand specific types of consumer techno-behaviours, working and learning preferences (where possible), and people’s movements/travelling. For example, data from 10 UK cities during the lockdown showed that air quality was significantly higher and anthropogenic noise was noticeably reduced (UKRI, 2020) due to travel restrictions, the slowdown of the economy, and the reduction of industrial waste and fossil fuel consumption (Rume and Islam, 2020). At the same time, while comparative studies showed some positive effects of individual practice on the environment such as e-commerce, more recent studies have shown that these effects tend to be negative or limitedly positive due to frequent goods return (Sievering, 2020), energy and packaging materials used in logistics networks (Matthews et al., 2001) and home-delivery model used (Gee et al., 2020; Siikavirta et al., 2002). Furthermore, the digitalization of society produces significant effects on both private and public (Gijzen, 2013; Schmidt and Cohen, 2013), generating higher attention to environmental issues (Embry et al., 2019; George et al., 2012).

Therefore, investigating digital environmental orientations should be a priority for those multi-level policy approaches that aim to promote sustainable uses of digital technologies. Private and public actors must direct the digital revolution in a way that is supportive of and essential to a sustainable future. The focus of the digital sustainable agenda should be on the individual, and policymakers at all levels need to take into account how the individual’s interactions with the economy, society, environment, and other factors all affect one another.

As suggested in the introductory sections of this work, the COVID-19 crisis might have created ‘opportunities’ thanks to both structural/contextual factors (such as more opportunities to work from home, restrictive rules and existing individuals’ socio, cultural and economic backgrounds) and individual agency towards the environment and digital technologies. Such opportunities might have helped individuals realise that the use of digital technologies can facilitate everyday life without affecting the environment. Looking at the specific digital environmental habitus of parents in England, this article suggested that parents’ education levels, gender, age and income play a role in increasing their awareness about the environmental-friendly use of digital technologies. These socioeconomic and sociodemographic variables need to be taken into consideration when formulating principles for a successful environmental policy or educational initiatives.

An interesting result is associated with the increasing digital–environmental orientation of male parents, who show a higher degree of self-reflection on the relationship between their use of digital technology and its effects on the environment. This suggests that the increased media coverage of CC-related issues in the United Kingdom coupled with the COVID-19 crisis might have activated processes of context-dependent reflexivity for this group. Although this is mere speculation, previous studies considered in the discussion of this work which were conducted outside the pandemic period, showed the opposite result, with mothers more worried about the environment. However, we also suggest that this might depend on the higher exposure to digital technologies given male parents’ jobs.

One of the most interesting results relates to the disconnection between CC beliefs and pro-environmental orientation in the use of digital technologies. This suggests that parents’ pro-environmental awareness and behaviours are not necessarily connected to their belief in CC. This aspect needs further exploration in qualitative terms to clarify this disconnection.

The quantitative and cross-sectional nature of this study did not allow for comparison with previous circumstances and evaluation of the potential effects of the digital technological acceleration caused by the pandemic compared with the previous conditions. Therefore, future research might need to focus on longitudinal panel design to look at the evolution of experiences. However, the COVID-19 pandemic accelerated the digitalisation of society and this is an urgent aspect to be considered in studies that intend to investigate pro-environmental behaviour, and environmental attitudes. The flexibility of habitus might, indeed, cause either a rejection of the original habitus or a transformative capacity to the new techno-digital context in light of contemporary environmental concerns. Policymakers should pay attention to direct the digital acceleration towards a pro-environmental attitude, which otherwise might be difficult to reorient once different constellations of habitus are consolidated. Specifically, to readdress the adoption and use of digital technologies in a more sustainable way, it might be useful to enhance the environmental and sustainability education throughout students’ journey. This might help to level up the parent’s socioeconomic and demographic differences and educate the future generation towards more pro-environmental and eco-friendly attitudes and behaviour.

At the same time, such an approach should pay attention to both, the existing economic, social and cultural stratification of digital–environmental habitus, and the stakes at play for people who engage with the evolving field of action.
